# Chikungunya patient transcriptional signatures faithfully recapitulated in a C57BL/6J mouse model

**DOI:** 10.3389/fimmu.2022.1092370

**Published:** 2022-12-12

**Authors:** Cameron R. Bishop, Felipe Ten Caten, Helder I. Nakaya, Andreas Suhrbier

**Affiliations:** ^1^ Department of Infection and Inflammation, Queensland Institute of Medical Research, Berghofer Medical Research Institute, Brisbane, QLD, Australia; ^2^ Pathology Advanced Translational Research Unit, Department of Pathology and Laboratory Medicine, Emory University School of Medicine, Atlanta, GA, United States; ^3^ Department of Clinical and Toxicological Analyses, School of Pharmaceutical Sciences, University of São Paulo, São Paulo, Brazil; ^4^ Global Virus Network (GVN) Center of Excellence, Australian Infectious Disease Research Centre, Brisbane, QLD, Australia

**Keywords:** Chikungunya, C57BL/6J, mouse model, RNA-seq, bioinformatics, arthritis

## Abstract

**Introduction:**

An adult wild-type C57BL/6J mouse model of chikungunya virus (CHIKV) infection and disease has been extensively used to study the alphaviral arthritic immunopathology and to evaluate new interventions. How well mouse models recapitulate the gene expression profiles seen in humans remains controversial.

**Methods:**

Herein we perform a comparative transcriptomics analysis using RNA-Seq datasets from the C57BL/6J CHIKV mouse model with datasets obtained from adults and children acutely infected with CHIKV.

**Results:**

Despite sampling quite different tissues, peripheral blood from humans and feet from mice, gene expression profiles were quite similar, with an overlap of up to ≈50% for up-regulated single copy orthologue differentially expressed genes. Furthermore, high levels of significant concordance between mouse and human were seen for immune pathways and signatures, which were dominated by interferons, T cells and monocyte/macrophages. Importantly, predicted responses to a series of anti-inflammatory drug and biologic treatments also showed cogent similarities between species.

**Discussion:**

Comparative transcriptomics and subsequent pathway analysis provides a detailed picture of how a given model recapitulates human gene expression. Using this method, we show that the C57BL/6J CHIKV mouse model provides a reliable and representative system in which to study CHIKV immunopathology and evaluate new treatments.

## Introduction

Chikungunya virus (CHIKV) is a mosquito transmitted alphavirus responsible for sporadic outbreaks of rheumatic disease, the most recent of which (2004-2019) resulted in >10 million cases in >100 countries on four continents (1). The disease is primarily associated with symmetrical polyarthralgia/polyarthritis, with other acute symptoms including fever, rash, myalgia and fatigue. A series of severe atypical manifestations are also recognized, with hospitalization rates ranging from 0.6% to 13% of cases and mortality rate estimates ranging from 0.024% to 0.7%. Chronic disease is also recognized and is primarily characterized by polyarthralgia/polyarthritis, but can also include depression, fatigue and alopecia, with ≈0.3-20% of patients reporting symptoms at 1 year post onset (1). Treatment for arthritogenic alphaviruses usually involves paracetamol (acetaminophen) and/or non-steroidal anti-inflammatory drugs, which can provide symptom relief (2). The rapid accumulation of cases, the occasionally high attack rates (up to 30-75% in certain populations) and the ensuing economic burden, have prompted widespread efforts to develop vaccines (3–7), antiviral therapies (8, 9) and more effective anti-inflammatory treatments (2).

Development of new interventions usually involves use of mouse models as early evaluation tools. Although a number of animal models have been described (10–12), the non-lethal, adult C57BL/6J mouse model of viremia and arthritic foot swelling (13–15) has been widely adopted internationally (16–23). This model has been used to investigate the virology, immunobiology and immunopathology of CHIKV infections (12, 14, 24–30). Given the considerable debate regarding how faithfully mouse models recapitulate the transcriptional responses seen in humans (10, 31–38), we recently developed a series of bioinformatic methods for investigating how well the transcriptional responses in a given mouse model mimic those seen in humans (39). For human CHIKV patients, RNA-Seq data is available for peripheral blood from acutely infected adults and children (40, 41). For the adult C57BL/6J mouse model, RNA-Seq data is available for both lymph nodes and arthritic feet during acute infection (14, 15). RNA-Seq provides an excellent tool for detailed characterization of any given mouse model to ascertain how faithfully it recapitulates human disease responses and to determine its reliability for evaluation and development of new interventions. Herein we use bioinformatic methods to evaluate the adult wild-type C57BL/6J mouse model of CHIKV infection and arthritic disease. Despite comparing transcriptional signatures from the peripheral blood of human patients with arthritic feet from infected mice, nearly half the genes significantly up-regulated by infection were shared between species. Pathway analyses also illustrated highly significant concordance for inflammatory responses, and predicted multiple drugs and biologics that have seen evaluation for CHIKV rheumatic disease. By these criteria, this mouse model of CHIKV shows a high level of consensus with human disease.

## Results

### RNA-Seq data sets for CHIKV infection in mice and humans

The mouse data sets were derived from pooled hind feet and inguinal lymph nodes of CHIKV and mock infected adult female C57BL/6J mice at 2 days post infected (dpi) (peak viremia) and 7 dpi (peak arthritic foot swelling) (15) (**Table 1**). The raw data (fastq files) were reanalyzed herein using STAR, RSEM and EdgeR, with a q<0.05 filter applied to provide Differentially Expressed Genes (DEGs) (**Tables S1A, D, G, J, M, P**). For each of these four DEG lists, a mouse-human orthologue DEG list (orthoDEGs) (**Tables S1B, E, H, K, N, Q**) and a single copy orthologue DEG list (scoDEGs) was generated (**Tables S1C, F, I, L, O, R**).

**Table 1:** Origins of human and mouse gene expression datasets.

Groups	NCBI BioProject	Infectedtissue	Control tissue	Library prep.	RNA-SeqPlatform	Notes
Mice Ft 2 dpi Ft 7 dpi LN 2 dpi LN 7dpi	PRJNA431476	Hind Ft Inguinal LN	Hind Ft Inguinal LN from mock infected mice	Poly-A selected	Illumina HiSeq 2000	2 dpi peak viremia 7 dpi peak arthritis
Human Children	PRJNA390289	Whole peripheral blood acute phase	Whole peripheral blood convalescent phase	Total RNA, ribo/globin RNA depleted	Illumina HiSeq 4000	Technical replicates collapsed
Human Adults	PRJNA507472	Whole peripheral blood acute phase	Whole peripheral blood healthy subjects	Total RNA, ribo/globin RNA depleted	Illumina HiSeq 1500	

The source and treatment of the RNA-Seq data sets from mouse and human studies. All fastq files were reanalyzed using STAR, RSEM and EdgeR to provide consistency across groups. Ft – feet, LN – lymph nodes, dpi days post infection.

The data sets from infected human adults were obtained from individual whole blood of adult CHIKV patients where blood was collected at or within 2 days of disease onset (n=30), with healthy adults providing control samples (n=20) (41) (**Table 1**). DEG, orthoDEG and scoDEG lists were generated (**Tables S1A–C**).

The data sets from human children were obtained from individual whole blood of acute pediatric CHIKV cases, with the controls being convalescent whole blood (40) (**Table 1**). Multi-Dimensional Scaling (MDS) and Molecular Degree of Perturbation analyses identified 12 and 20 samples, respectively, as outliers (**Figure S1**), which were removed leaving 140 accessions (n=71 after collapse of technical replicates) for n=35 infected and n=36 convalescent samples (**Table 1**). DEG, orthoDEG and scoDEG lists were generated (**Tables S1D–F**).

### Single copy orthologues, principle component analysis and hierarchical clustering

**Figure 1:**
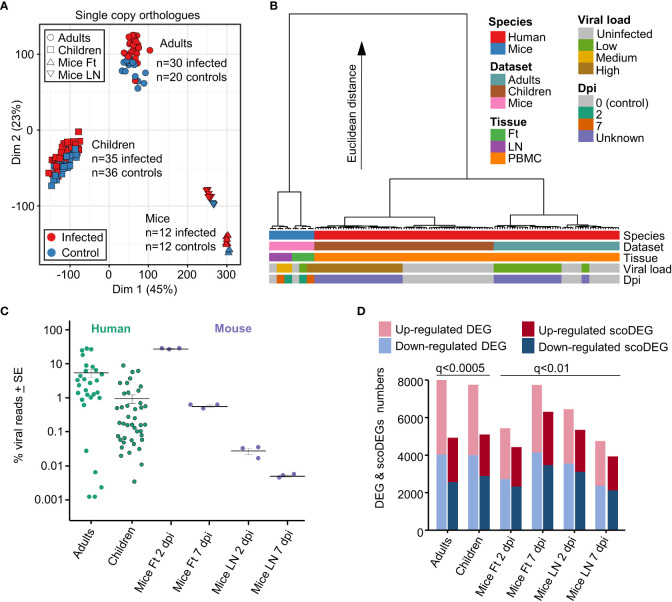
Principal component and hierarchical cluster analyses, viral loads, DEGs and scoDEGs. **(A)** Principal component analysis (PCA) plot (PC1 - Dim1, PC2 - Dim2) for all accessions using all single copy orthologues. **(B)** Hierarchical cluster analysis using log2 TMM-normalized read counts of the top 500 single copy orthologues ranked by PC1 and PC2 loadings. **(C)** The number of viral reads in each accession expressed as a percentage of the total number of reads aligning to protein coding genes in the human or mouse genome in the same sample. Cross-bars represent the mean for each group. **(D)** Histogram showing the number of DEGs and scoDEGs for each group after application of the indicated significance cutoffs (q, FDR).

When expression data from all single copy orthologues from all samples were displayed on a MDS plot, a clear separation emerged between Adults, Children and Mice samples, with differences between these 3 groups much larger than differences between infected and uninfected samples (**Figure 1A**). The source of the samples (Adult, Children or Mice), rather than the presence of a CHIKV infection, thus had the dominant role in determining the gene expression profiles.

**Figure 2:**
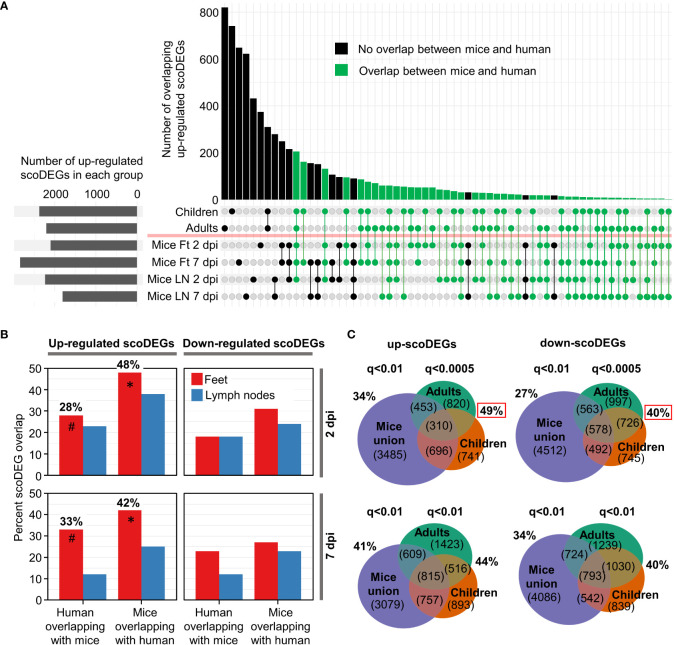
ScoDEG overlaps. **(A)** Upset plot of overlaps of upregulated scoDEGs (from **Figure 1D**). The overlap between the union scoDEGs (scoDEGs from all mouse groups) and human adults and children. **(B)** The percent overlap between scoDEGs for all groups. Corresponding Euler diagrams in **Figure S4**. * Highest overlaps, percent of up-regulated scoDEGs identified in Mice Ft 2 and 7 dpi that were also identified in human groups. ^#^ For the same groups, but the percentage of up-regulated scoDEGs identified in humans that were also identified in Mice Ft 2 and 7 dpi. **(C)** Top two Euler diagrams; scoDEG overlaps using the FDR (q) cutoffs described in **Figure 1D**, but where all scoDEGs from all mouse groups are concatenated (union scoDEGs). Red boxes; overlap of scoDEGs identified in humans that were also identified in any mouse group. Bottom two Euler diagrams; same as above except using a cutoff of q<0.01 for scoDEG list generation for all groups.

To more fully understand the main sources of variation between the groups, a hierarchical clustering analysis was undertaken (**Figure 1B**). The largest contribution to variation in mRNA expression patterns between the groups was species (**Figure 2B**, blue & red), consistent with previous analyses of COVID-19 mouse models (39). Thereafter, the largest contributors to variation were mouse tissue type (feet vs. LNs) (**Figure 2B**, Tissue, purple & green) and Adults vs. Children (**Figure 2B**, Dataset, brown & pale blue). Viral load (see below) and days post infection provided comparatively minor contributions to the Euclidian distance, consistent with **Figure 1A** and previous analyses (39).

### Blood transcription modules and peripheral blood lymphopenia

The sizable separation between Adult and Children (**Figure 1A**) is perhaps surprising, although CHIKV arthropathy is generally viewed as less severe in children when compared with adults (1, 42). Early innate responses are also reported to be more vigorous in children (43) and dominated by monocyte driven responses (40). Consistent with the latter findings, Gene Set Enrichment Analysis (GSEA) using Blood Transcription Modules (BTMs) (44) and gene lists ranked by fold change, revealed more significant GSEAs for Children than for Adults, often for BTMs associated with monocytes (**Figure S2**). Significant GSEAs for BTMs associated with antiviral responses were also evident in both gene sets, and importantly, both gene sets showed signatures consistent with lymphopenia (**Figure S2**). Transient peripheral blood lymphopenia is a known feature of acute CHIKV infections in primates (45–47).

### Comparable viral loads for mice feet and human groups

The reference genomes for mouse and human were augmented to include the CHIKV viral genome (Reunion Island isolate, LR2006_OPY1, GenBank KT449801), with viral read counts providing quantitation of the viral RNA load within each sample. Viral read counts are presented as a percentage of read counts aligning to mouse or human protein coding genes, with the results illustrating that both mouse and human groups covered an overlapping ≈4 log range (**Figure 1C**). The mean percent viral read counts for human samples was comparable to those seen in mice feet, with LNs showing 2-3 logs lower viral reads (**Figure 1C**).

As the latent period for CHIKV in humans is 2-6 days, both human and murine studies represent samples taken within a week of infection and encompass both the period of acute viral replication and the, often abrupt and fulminant, onset of rheumatic disease (13, 40, 41, 48). Onset of disease (usually fever and arthropathy) in humans occurs around the time of peak viremia (49, 50). Blood for both human data sets was obtained within 2 days of symptom onset (40, 41). The lower viral loads in Children (**Figure 1C**) are likely associated with the faster clearance of viremia as a result of the aforementioned stronger early innate immune responses (43).

### DEGs and scoDEGs

The number of biological replicates for the human studies were much higher than for the mice study [which pooled RNA from 4 mice for each of the 3 replicates (15)] (**Figures 1A, C**). As a result, DEG numbers were substantially higher for the human studies. The q value (FDR) cutoff was thus adjusted so (i) that human and mouse groups had broadly similar number of scoDEGs, allowing human-mouse comparisons without introducing bias from different gene set sizes, and (ii) that DEG numbers were below 8000, the limit for Ingenuity Pathway Analysis (IPA) (see below) (**Figure 1D**).

When the human DEG lists were curated to contain only scoDEGs, nearly a third of the DEGs were lost (**Figure 1D**). These lost human non-scoDEGs were analyzed further to ensure that important information was not lost from this process. Nearly 40% of non-scoDEGs had an ENS prefix (Ensembl) rather than a HUGO ID (**Figure S3A**), and primarily represent long non-coding RNA and pseudogenes. These genes also had, on average, lower read counts (**Figure S3B**). Finally, IPA analysis of the non-scoDEGs provided very similar UpStream Regulator (USR) z-scores as those identified for scoDEGs (**Figure S3C**). Thus removal of non-scoDEGs from the human DEG lists would appear not to excessively remove important information.

### Overlap between mouse and human scoDEGs

For the up-regulated scoDEGs described in **Figure 1D**, the scoDEG overlaps between the groups is illustrated in an Upset plot (**Figure 2A**). For many scoDEGs there was no overlap between mouse and human groups (**Figure 2A**, black bars and lines/circles). Using a series of Euler diagrams (**Figure S4**), the best scoDEG overlaps between species was seen for mice feet were 48% (2 dpi) and 42% (7 dpi) of up-regulated scoDEGs were also identified in human blood (**Figure 2B**, asterisks). However, for the same groups, only 28% and 33% of scoDEGs identified in humans were up-regulated scoDEGs in mice feet for 2 and 7 dpi, respectively (**Figure 2B**, hash symbols). Overlap percentages were lower for down-regulated scoDEGs, and lower again for LNs (**Figures 2B**, **S4**). That down-regulated scoDEGs show lower levels of overlap may reflect infection of different cell types (e.g. fibroblasts in feet (1) vs. monocytes in blood (51)), but likely also reflects CHIKV-induced lymphopenia in human peripheral blood (**Figure S2**) versus arthritic infiltrates in mice feet (13).

The aforementioned analysis (**Figure 2B**, hash symbols) argues that the majority of the scoDEGs identified in peripheral blood of humans were not scoDEGs in mice. However, when the union of mouse scoDEGs was compared with humans, percentage overlaps increased, with 49% and 40% of scoDEGs identified in humans seen in at least one mice group, for up and down-regulated scoDEGs, respectively (**Figure 2C**, red boxes). These percentages did not increase when the FDR (q) values were changed to q<0.01 (for DEG cutoff) for the human groups (**Figure 2C**, bottom Euler diagrams).

Given the different source of infected material (feet for mice and peripheral blood for humans) (**Table 1**), overlaps of nearly 50% for up-regulated scoDEGs (**Figure 2B**, asterisks; **Figure 2C**, red box) might be considered a relatively high level of consensus between species (39).

### Reciprocal GSEAs show species concordance

Reciprocal GSEAs were performed using the gene lists ranked by fold change and the DEG sets for each group. For mouse/human single-copy orthologues with different HUGO IDs between species (≈8%), the mouse gene symbols were changed to their orthologous human equivalent in the DEG sets and the orthologue lists. This allowed GSEAs to be undertaken for mouse vs. human orthologue sets. A separate GSEA was performed for each up- and down-regulated DEG set.

**Figure 3:**
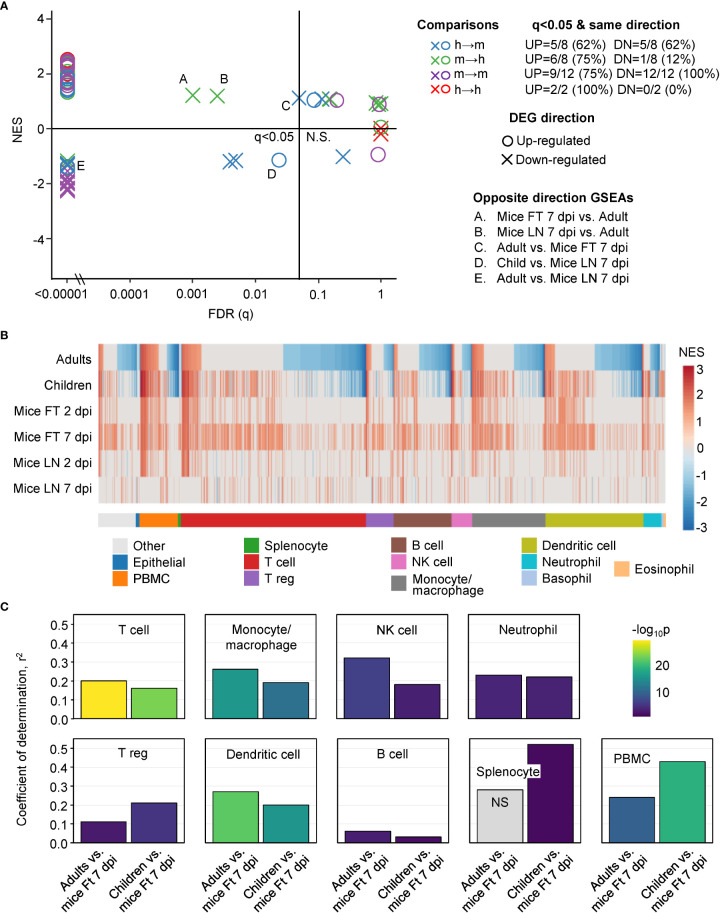
GSEAs between groups and using ImmuneSigDB gene sets. **(A)** Reciprocal GSEAs using genes lists ranked by fold change and up- and down-regulated DEG lists from each group. Most GSEAs were significant (left of q=0.05 line), with the number of significant GSEAs over the number of GSEAs provided top right (q<0.05 & same direction). Five GSEAs gave opposite direction; e.g. up-regulated orthoDEG sets significantly enriched in the down-regulated ranked gene list (Opposite direction GSEAs). **(B)** GSEAs using up-regulated gene sets from ImmuneSigDB and gene lists ranked by fold change for all the groups. GSEAs significant in at least one group are segregated by cell type (mentioned in the ImmuneSigDB annotation) and ranked by Adults. Blue represents up-regulated ImmuneSigDB gene sets that are enriched in the down-regulated genes in the ranked gene lists. **(C)** For the GSEAs shown in b, NES for Adults vs. Ft 7 dpi and Children vs. Ft 7 dpi were plotted for the indicated cell types and Pearson correlation coefficients and significance values obtained.

The human up-regulated DEGs were significantly enriched with positive Normalized Enrichment Scores (NES) in 5/8 of the ranked mice gene lists (**Figure 3A**, blue circles; **Table S2**). The up-regulated DEGs from mice were significantly enriched with positive NES in 6/8 of the ranked human gene lists (**Figure 3A**, green circles; **Table S2**). The numbers were 5/8 (human-mouse) and 1/8 (mouse-human) for down-regulated DEGs and negative NES scores (**Figure 3A**, blue and green crosses; **Table S2**). Five significant GSEAs found signatures for up-regulated DEGs in the down-regulated genes or down-regulated DEGs in the up-regulated genes (**Figure 3**, opposite direction GSEAs); a result that likely reflects, in part, the aforementioned leukopenia. Thus although some incongruities were evident, these GSEA results argue that genes significantly up-regulated in mice, often showed significant enrichment in human ranked gene lists and *vice versa*.

### GSEAs using ImmuneSigDB gene sets

ImmuneSigDB is a compendium of 5219 immunology-specific gene sets that can be used to interrogate mouse and human ranked gene lists using GSEAs to identify immune signatures (37). A ranked gene list was used as input for each GSEA. As before, mouse/human single-copy orthologues with HUGO IDs that differed between species were changed in the mouse lists to their orthologous human equivalents. GSEAs using up-regulated ImmuneSigDB gene sets that were significantly enriched in at least one ranked gene list were used to compare groups (n=1616) (**Table S3**).

The NES are plotted for GSEAs that reached significance (q<0.05) in at least one group, with the NES ranked by Adults (**Figure 3B**). The results were also grouped by cell type according to the specific cell type mentioned in the ImmuneSigDB gene set annotations (**Figure 3B**) (37, 39). GSEAs with negative NES were prominent in the human groups (**Figure 3B**, Negative NES, Blue), an observation consistent with the peripheral blood lymphopenia (45, 52) (**Figure S2**). The inferred lymphopenia signature was less prominent in Children (**Figure 3B**, Negative NES, Blue), perhaps associated with the lower viral loads (**Figure 1C**). As might be expected, lymphopenia was not a feature of mouse groups, as arthritic feet and LN were sampled, rather than peripheral blood (1, 53–55). This GSEA incongruity (**Figure 3B**, Negative NES, Blue) is thus more likely due to the source of the samples, rather than representing a difference between species.

For the GSEAs with positive NES scores there was considerably more concordance between mouse and human groups, with the exception of Mice LN 7 dpi (**Figure 3B**, Red). Mice LN 7 dpi showed the lowest viral loads of all the groups (**Figure 1C**), with the viremia usually no longer detectable by 6 dpi in this model, although high tissue viral titers remain in feet (13, 15). The larger number of ImmuneSigDB GSEAs with positive NES scores for Mice Ft 7 dpi (**Figure 3B**, Red), is likely due to the overt foot swelling and pronounced, CD4 T cell-dependent, monocyte/macrophage dominated, inflammatory infiltrate seen in feet at this time (13, 55–57).

For the main cell types in **Figure 3B**, Pearson correlations were determined for the NES scores for Adults vs. Mice Ft 7 dpi and Children vs. Mice Ft 7 dpi (**Figure 3C**). Correlations were highly significant for the main cell types involved in CHIKV arthritis; T cells (56–58), monocyte/macrophages (45, 51, 55) and NK cells (59), and to a lesser extent neutrophils (54, 60), regulatory T cells (61, 62) and dendritic cells (13) (**Figure 3C**). Coefficients of determination (r^2^) were generally not so high (**Figure 3C**), likely reflecting the influence of the aforementioned lymphopenia.

Despite the confounding effect of lymphopenia vs. arthritic infiltrates, overall these analyses argue that during acute CHIKV infections, the dominant immune responses up-regulated in peripheral blood in humans are surprisingly similar to the dominant immune responses up-regulated in arthritic feet in mice.

### High species concordance for inflammatory pathways

**Figure 4:**
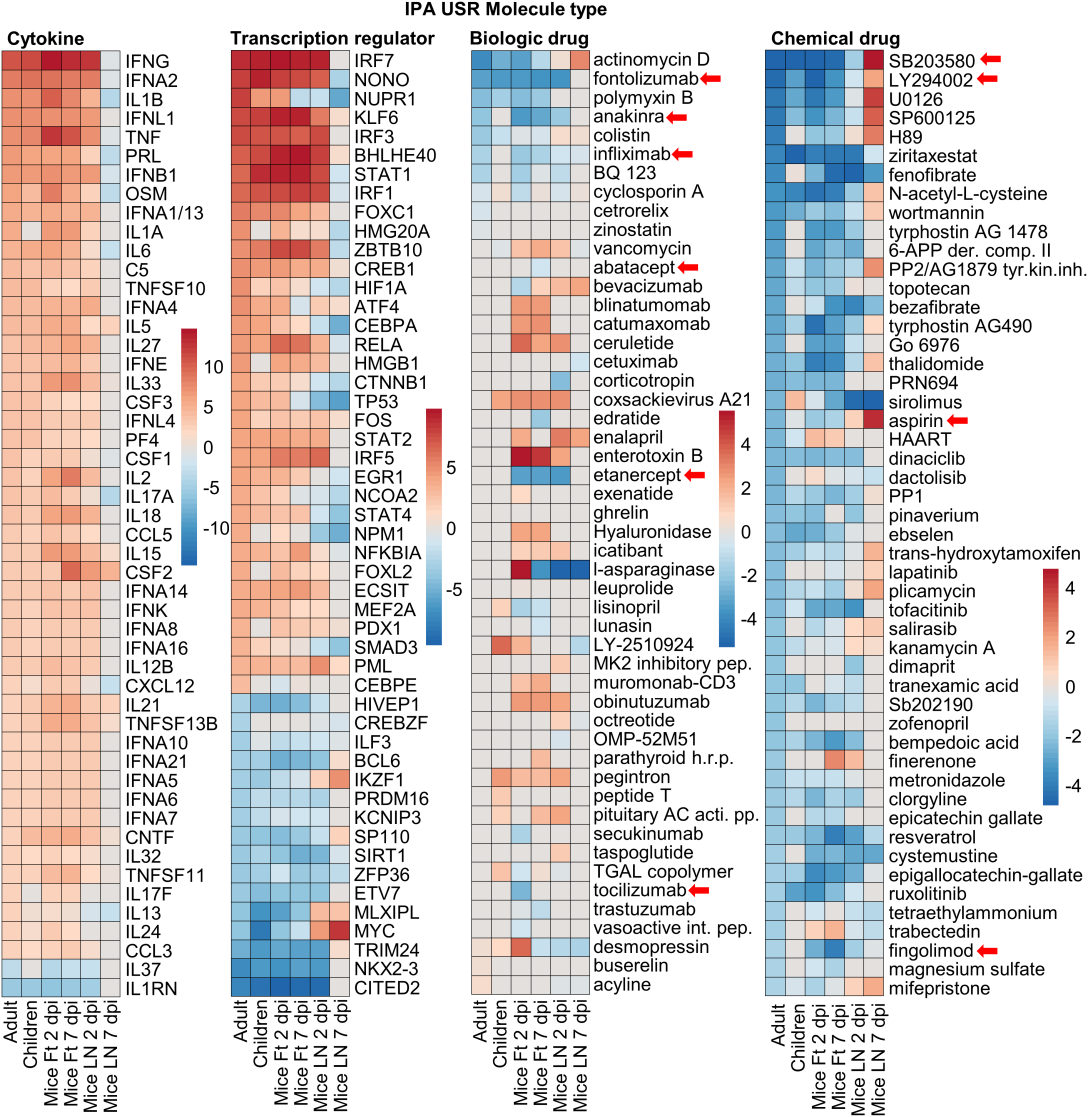
IPA USRs heatmaps. The DEG lists for all groups were analyses by IPA and the top 50 USRs (significant in at least 1 group) plotted by molecule type and ranked by USR z-scores for Adults. Cytokine and Transcription regulator – USRs for Adults ranked by absolute z-score. Biologic and Chemical drug – ranked from highest negative z-score (most potent predicted inhibitor). Red arrows indicates compounds referred to in the text. Abbreviations: AC acti. pp - adenylate cyclase-activating polypeptide; intest. pep. intestinal peptide; APP der. comp. II - 6-aminopyrazolopyrimidine derivative compound II; h.r.p.- hormone related protein; HAART - highly active antiretroviral therapy; tyr.kin.inh. tyrosine kinase inhibitor.

IPA accepts both human and mouse gene IDs, and the Up-Stream Regulator (USR) feature identifies the most likely up-stream causes (by p values and z-scores) of the transcriptional changes seen in any given DEG list. Using the DEG lists (**Figure 1D**) significant (p<0.05) IPA USRs were identified for each group (**Table S4**). USR are grouped by Molecule type, and for Cytokine and Transcription regulator USRs, the top 50 USRs by absolute z-score are shown ranked by Adults (**Figure 4**). A high level of concordance is apparent across species, with the exception of LN 7 dpi (**Figure 4**, Cytokine, Transcription regulator). The general congruence between Adults and Children (**Figure 4**) is consistent with a previous study showing a common pattern of immune signatures associated with CHIKV infections in humans across age groups (43). The contention is further supported by plots showing relatively minor differences in USR z-scores for Adults versus Children (**Figure S5**).

Most of the top cytokines and transcriptional regulators (**Figure 4**) are well described in the CHIKV literature, and as expected are dominated by interferon (IFN) and Th1 responses (1, 15, 40, 41, 54, 60, 63–66). Perhaps surprising is the absence of CCL2/CCR2 as an USR, even though CCL2 is a highly significant DEG, and is well described for CHIKV infections (45, 55, 67). Conceivably, this is an under-annotation issue for IPA.

### Species concordance for biologic and chemical drug USRs

A key question is whether new treatments evaluated in mice might provide reliable insights into therapeutic activity in humans. Biologic and Chemical drug USRs (identified by IPA as above) were ranked by negative NES (most likely to inhibit) in Adults (**Figure 4**, Biologic drug, Chemical drug). Many of the drugs identified herein have been the subject of independent evaluations in mice and/or humans (**Figure 4**, red arrows). Only very early subcutaneous edema is reduced in feet of IFNγ-/- mice, with marginally increased viremia (15), with the anti-IFNγ biologic, fontolizumab, to the best of our knowledge not evaluated for any alphavirus. Targeting IL1B with Anakinra shows some benefit in mice (68), but Anakinra has not been evaluated in CHIKV patients. Experience with anti-TNF biologics, such as infliximamb (anti-TNF monoclonal antibody) and etanercept (TNF receptor fusion protein), have been reported in CHIKV patients (69, 70) and may reduce duration of symptoms (71). Mouse experiments are limited to a weanling mouse model of Ross River virus (RRV) where etanercept treatment was started 1 day after infection (i.e. before antibody production), and caused exacerbated disease and lethality (72). The anti-CD80/CD86 monoclonal, abatacept, has shown promise for CHIKV arthritis (that peaks 6/7 dpi) in mice (73), but IPA analysis of human expression data from peripheral blood did not predict efficacy in CHIKV patients (**Figure 4**, abatacept z-score = 0). Bone loss (not generally a significant feature of CHIKV disease in humans) is reduced by anti-IL-6 antibody in the weanling mouse model of RRV (74). However, limited studies on the anti-IL6 receptor antibody, tocilizumab, have not shown benefit in CHIKV patients (71), nor does the IPA analysis predict efficacy (**Figure 4**, Biologic drug).

The top scoring Chemical drugs are SB203580 (Adezmapimod, a p38 MAP kinase inhibitor) and LY294002 (PI3K inhibitor) (**Figure 4**, Chemical drug), with both shown to have some anti-alphaviral activity *in vitro* (75, 76). Importantly, aspirin, a well annotated representative of non-steroidal anti-inflammatory drugs (NSAIDs) is identified across species, with NSAIDs the mainstay of treatment for alphaviral arthropathies (48, 77). Fingolimod (a sphingosine-1-phosphate receptor modulator that sequesters lymphocytes in lymph nodes) has shown efficacy against CHIKV arthropathy in mice (78). MCC950 is absent, despite activity in mice (27), as no annotation exists in IPA for this NLRP3 inhibitor. Also absent are disease modifying anti-rheumatic drugs (DMARDs) (e.g. methotrexate and sulfasalazine), with these well annotated within IPA; however, despite a number of studies, no clear benefit has been established in patients with acute CHIKV (77). Failure to formally exclude CHIKV patients that may also have underlying autoimmune arthritides may have complicated a number of DMARD studies (1). Corticosteroid use (79, 80) was similarly not highlighted by our analyses.

Taken together these analyses point to a high level of conservation for predicted drug sensitivities between CHIKV patients and the adult C57BL/6J CHIKV mouse model, with many of the drugs that were identified (**Figure 4**, red arrows) also the subject of investigations in mice and CHIKV patients.

### Human USR pathways correlate better with mouse feet than mouse LNs

**Figure 5:**
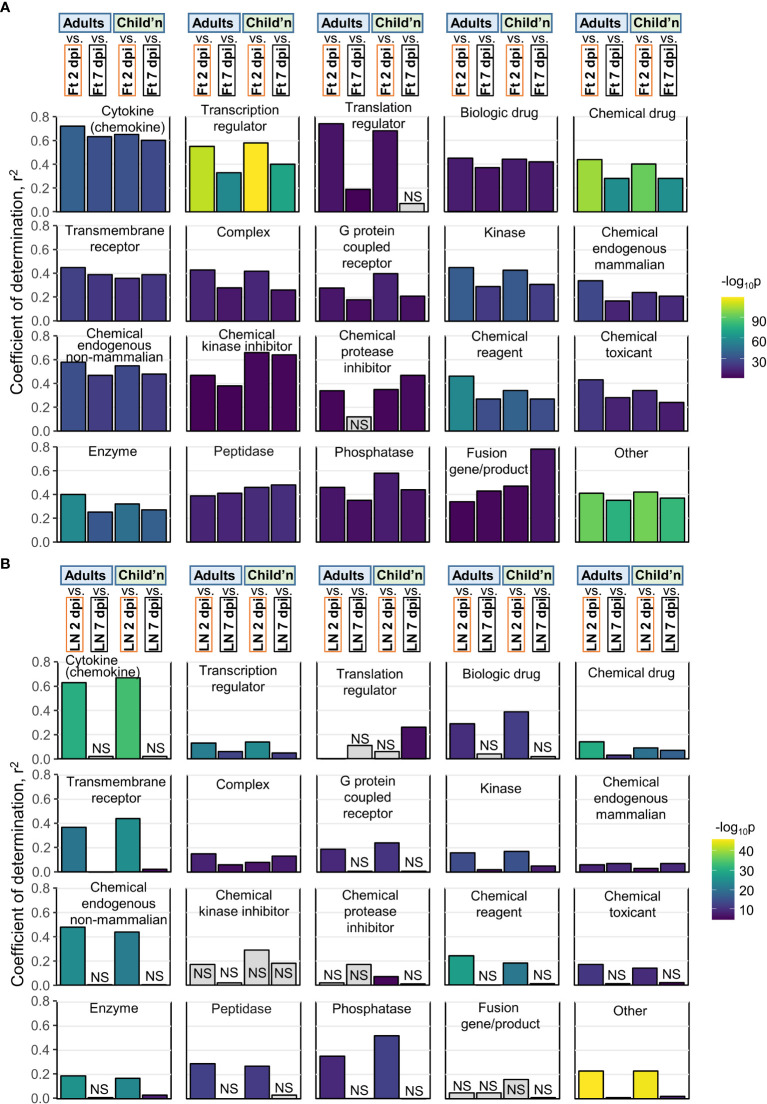
Pearson correlations for IPA USRs. **(A)** The DEGs from Adults, Children, mice Ft 2 dpi, and mice Ft 7 dpi were analyzed by IPA to provide z-scores for significant USRs (p<0.05). The USRs were then grouped by Molecule type and Pearson correlations undertaken. For example, top left, Cytokine, compares Adults Cytokine USR z-scores with mice feet 2 dpi and 7 dpi Cytokine USR z-scores (correlation plot shown in **Figure S6A**). **(B)** As for a, but correlations with mice LN 2 and 7 dpi.

Pearson correlations were undertaken for pairwise comparisons of z-scores across all USR molecule types for Adults and Children versus mouse groups. Coefficients of determination (r^2^) for human versus mice feet (**Figure 5A**; **Figure S6**) were nearly always higher than for human versus mice LNs (**Figure 5B**), with LN 7 dpi again showing very poor correlations (**Figure 5B**, LN 7 dpi). Thus for acute CHIKV infections, arthritic feet from mice, rather than LNs, show a higher level of congruence with human peripheral blood across multiple pathways. This may reflect the fact that leukocytes, including antigen-specific T cells and other lymphocytes, extravasate from the peripheral blood to the sites of infection (joints) (81) and become responsible for the CHIKV inflammatory arthropathy (1).

### Specificity of CHIKV signatures

**Figure 6:**
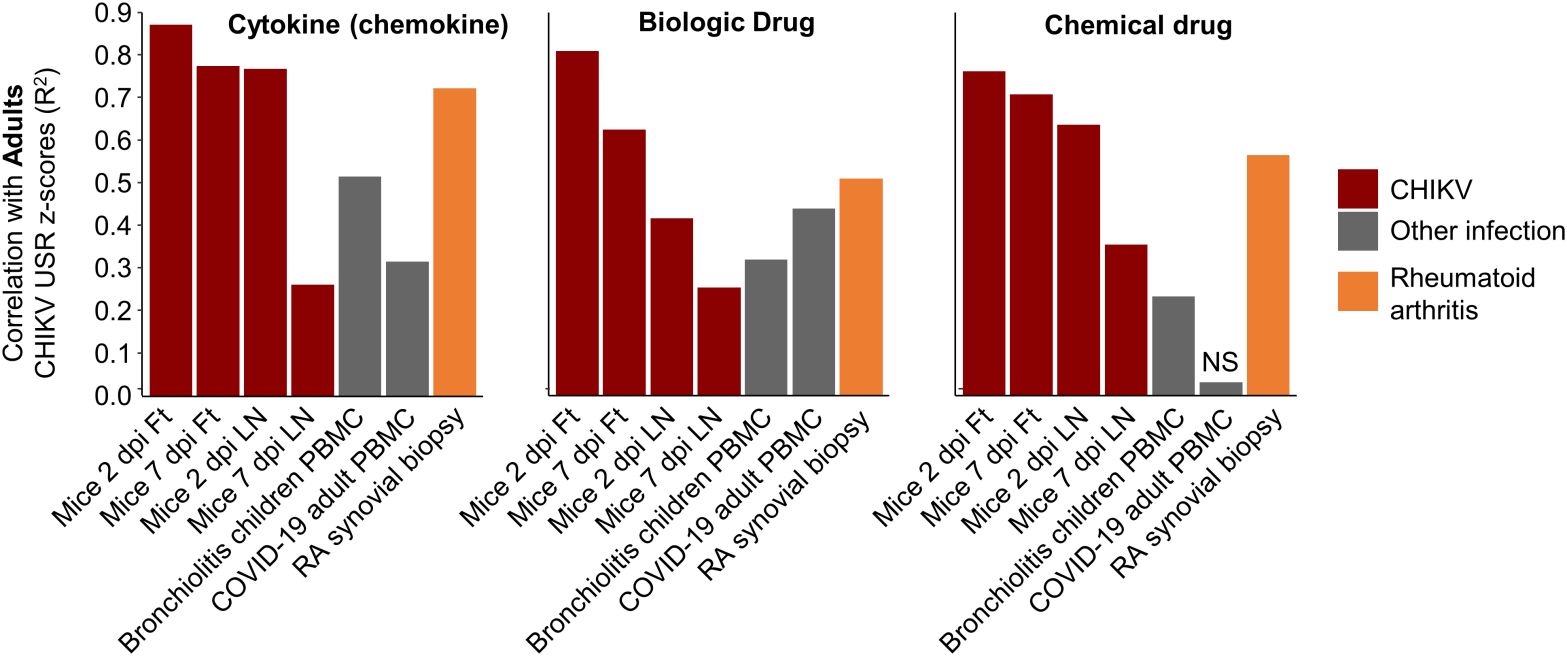
Pearson correlations for IPA USRs with other infections and rheumatoid arthritis. The DEGs from whole blood of CHIKV-infected Adults were analyzed by IPA to provide z-scores for significant USRs and USRs for (Molecule type) Cytokine, Biologic drug and Chemical drug. These z-scores were compared by Pearson correlations with z-scores from peripheral blood mononuclear cells (PBMC) from children with bronchiolitis and Adults infected with SARS-CoV-2, as well as synovial biopsies from rheumatoid arthritis (RA) patients. Coefficients of determination (r^2^) are plotted.

A potential criticism of this type of interspecies comparison is that the signatures being compared are simply a generic reflection of infection and inflammation. Therefore, by reanalyzing publically available RNA-Seq data, we compared the degree of correlation of pathway signatures between CHIKV-infected humans and CHIKV-infected mice with those of CHIKV-infected humans and two non-CHIKV sources of human inflammatory disease. Pearson coefficients of determination comparing Cytokine USR z-scores from (i) PBMCs of adults with acute CHIKV infection versus feet of CHIKV-infected mice, were considerably higher than (ii) PBMCs of adults with acute CHIKV infection versus PBMCs of children with acute bronchiolitis (82) (**Figure 6**; Cytokine). A similar result was obtained for comparisons of Cytokine USR z-scores from PBMCs of adults with acute CHIKV infection versus PBMCs of adults acutely infected with SARS-CoV-2 (83) (**Figure 6**). Using Cytokine USR z-scores from peripheral blood of Children with acute CHIKV infections instead of Adults, did not significantly change these results (**Figure S7A**). Thus the best determinant of congruence of cytokine signatures was CHIKV, rather than infection, species or tissue.

Given our previous work on the similarities between rheumatoid arthritis (RA) in humans and CHIKV arthritis in mice (based on microarray studies) (56), we accessed publically available RNA-Seq data from synovial biopsies of RA patients (compared with healthy controls), generated DEGs, obtained IPA USR z-scores and used these for the same correlations. Pearson coefficients of determination were higher than those seen for bronchiolitis and SARS-CoV-2, although did not reach the r^2^ values seen for correlations with mice groups (**Figure 6**). These results support the view that arthritic signatures are observable in peripheral blood (84), and reaffirm the view (also seen in **Figure 4**, drug) that drugs used in the treatment of RA may find utility in the treatment of alphaviral arthritides (56) (**Figure S7B**).

### C57BL/6J versus C57BL/6N mice

The C57BL/6J mouse, bred at the Jackson Laboratory, is arguably the most commonly used mouse strain in medical research. There are, however, multiple C57BL/6 mouse strains (85), including the original C57BL/6N mice from which many knock-out mice are derived. The latter have a number of genetic differences from C57BL/6J mice (86, 87) and show an ameliorated CHIKV arthropathy, in part due to the presence of an intact Nicotinamide Nucleotide Transhydrogenase (*Nnt*) gene (14). MDS plots illustrate all mice infected feet groups cluster together closely when Adults and Children are included, illustrating that differences in transcriptional profiles between Adults and Children is much greater than between mouse strains (**Figure S8A**). When Children are removed, feet from infected C57BL/6N mice clearly segregate from C57BL/6J mice, although the Dim2 axis (on which segregation is evident) accounts for only 7% of the total variance (**Figure S8B**). Only when C57BL/6N are analyzed by themselves can the influence of *Nnt* be seen on the MDS plot (**Figure S8C**); the influence was explained in greater detail previously (14). This expression profiling argues that C57BL/6N mice do not ostensibly provide a better model of CHIKV, when compared with C57BL/6J mice, with overall differences between mouse strains actually quite minor when compared with differences between humans.

## Discussion

Herein we provide a series of bioinformatic approaches that illustrate a validation process for the adult C57BL/6J mouse model of CHIKV infection and disease using publically available RNA-Seq data sets. Overlap of up-regulated scoDEGs reached nearly 50%, despite the fact that human data sets were derived from peripheral blood (with associated lymphopenia) and murine data sets were obtained from arthritic feet (that contain inflammatory infiltrates). This scoDEG overlap is higher than the ≈15-35% seen for a similar bioinformatic study for SARS-CoV-2 infections in humans and mouse lung tissues (39). For the latter study, only fixed human lung tissues were available and days post disease onset for the COVID-19 patients was also not known, perhaps contributing to a lower level of overlap. Either way, both studies showed a high level of congruence for inflammatory and immune pathways between mice and humans. Importantly, the predicted behavior of a range of anti-inflammatory CHIKV treatments also showed a high level of concordance between humans and mice, arguing that the CHIKV mouse model represents a reliable and representative model in which to evaluate anti-inflammatory interventions.

A limitation of this study is that we were unable to compare mouse and human studies of chronic CHIKV disease. Chronic CHIKV disease (primarily arthropathy) is well described in humans (88, 89); however, RNA-Seq data is not available and establishing cohorts of chronic CHIKV patients free of other underlying (often autoimmune) rheumatic conditions is often difficult (1, 90). RNA-Seq data for 30 dpi is available for the C57BL/6J mouse model, but this might be viewed as post-acute rather than chronic (91), and largely only portrays a diminution of acute responses (15). We were also unable to address severe manifestations of CHIKV, which can include lethality, usually in very young and elderly patients (1, 92). Although lethal mouse models of CHIKV exist, they involve use of young mice or GMO mice defective in the type I IFN pathway (64, 93, 94). RNA-Seq data is currently not publically available for such mouse models, nor for CHIKV patients with lethal outcomes. Another limitation is that current bioinformatic approaches are unable reliably to predict treatments that might exacerbate inflammation (54, 55) or compromise anti-viral immunity (47, 72, 95, 96). However, with respect to the latter, in most settings, diagnosis of alphaviral arthritis involves paired serology (97), with treatment thus generally initiated after protective anti-viral antibodies have been generated. Finally, there are many biological processes that are cell-type specific and/or for which pathway annotations are poor or not readily available. In such cases the whole tissue RNA-Seq approach and the pathway analysis tools used herein may often be uninformative. An example would be the interplay between apoptosis and autophagy (98, 99), where species-specific response have been reported (100).

Understanding how a mouse model does or does not recapitulate certain responses seen during human disease is often important for assessing the validity of mouse experiments for understanding aspects of infection and disease, and for evaluation of particular interventions. Given there are published reports of mouse models recapitulating responses to infection and inflammation both well (33, 39, 101) and poorly (10, 34, 102), RNA-Seq and subsequent pathway analyses provide a useful strategy for determining exactly how well any given model recapitulates specific gene expression patterns seen in human patients. The adult wild-type C57BL/6J mouse model of CHIKV shows up-regulation of many genes that are up-regulated in humans, as well as showing a high level of concordance for immune and inflammation pathways, arguing that it overall provides a highly representative model of human disease.

## Materials and methods

### Gene expression analysis

Raw sequence reads (see **Table 1**) were accessed from the National Centre for Biotechnology Information Sequence Read Archive using Aspera (IBM). Quality control of sequence reads was performed using FastQC v0.11.9 (103). Reads were quality-trimmed with a minimum Phred score cutoff of Q20, and size-selected with a minimum length of 36 nucleotides, using Trimmomatic v0.36 (104). Trimmed reads were aligned to either the GRCm39 vM26 or GRCh38 v37 reference genome for mouse and human datasets, respectively, using STAR aligner v2.7.1a (105). The KT449801.1 CHIKV genome (58) was appended to each reference genome prior to read-alignment to allow quantitation of viral reads. Quantitation of viral reads was performed using primary proper paired reads, with Samtools v1.9 (106). Gene expression was calculated using RSEM v1.3.1 (107). Differentially expressed genes were identified using EdgeR v3.34.0 (108) in R v4.1.0 (109). Mouse-human orthologues and single-copy mouse-human orthologues were obtained from the Ensembl database using BiomaRt v2.48.2 (110) in R. A proportion of mouse-human single-copy orthologues (~8%) have HUGO IDs that differ between species. Therefore, all mouse samples had the HUGO IDs of these genes converted to the human equivalent prior to performing cross-species comparisons. Gene expression profiles were visualized across all samples using multi-dimensional scaling and hierarchical clustering analysis in R. Data were plotted using R packages, Eulerr v6.1.0 (111), ggplot2 v3.3.5 (112), ggpubR v0.4.0 (113), ComplexUpset v1.3.3 (114), PCAtools v2.4.0 (115), and RColorBrewer v1.1 (116).

### Reciprocal gene set enrichment analysis

An up- and a down-regulated DEG set was created for each group. A minimum absolute log_2_ fold-change cutoff was determined separately for each group by ranking all DEGs by absolute log_2_ fold-change (i.e. ignoring directionality) and then calculating the 50^th^ percentile. For a DEG to be included in the set, it had to have either a log_2_ fold-change greater than the cutoff (up-regulated DEGs) or lower than the negative cutoff (down-regulated DEGs). A log_2_ fold-change ranked gene list was produced for each group using EdgeR as described above. Pairwise Gene Set Enrichment analyses were performed using the up- and down-regulated DEG sets and ranked gene lists, with GSEA v4.1.0 (117) with 100 permutations and the ‘no_collapse’ setting. HUGO IDs of single-copy orthologues were standardized between species as described above.

### ImmuneSigDB gene set enrichment analysis

The ImmuneSigDB v7.4 gene set collection composed of 5219 immune-related gene sets was obtained from the Molecular Signatures Database. For each dataset and time point a GSEA was performed to test for enrichment of up-regulated ImmuneSigDB gene sets within the log_2_ fold-change ranked gene lists produced using EdgeR as described above. Data were visualized using pheatmap v1.0.12 (118) in R.

### Pathway analysis

Pathway analysis was performed using Ingenuity Pathway Analysis (IPA) v65367011 (Qiagen) with default settings, using log_2_ fold-changes of DEGs produced using EdgeR as described above. Data were visualized using pheatmap v1.0.12 in R.

### Comparison with non-CHIKV disease

The NCBI Bioprojects database was searched for projects containing the terms RNA-Seq, Homo sapiens, inflammation, SARS-CoV-2, PBMC, rheumatoid arthritis, and synovial. From the results of the search, three projects were chosen according to the following criteria: they were each associated with at least one peer-reviewed publication, had adequate controls (i.e. uninfected healthy individuals), and were generally similar to the CHIKV projects with respect to tissue, sequencing platform and method (i.e. bulk RNA-Seq). For the bronchiolitis dataset, all child samples were retained, and all infant samples were removed. For the SARS-CoV-2 dataset, all samples from healthy patients, and all samples from infected patients at zero days post testing PCR-positive, were retained. For the rheumatoid arthritis dataset, 20 age matched healthy and ‘established’ rheumatoid arthritis samples were retained. Differential expression was measured as described above, with a q-value cutoff of 0.05. Pathway analysis was performed using IPA as described above.

### Statistics

Statistics were performed using R version 4.1.0. For gene expression and pathway enrichment data a Pearson’s correlation test was used in accordance with the central limit theorem. Correlations with a p-value < 0.05 were considered significant.

## Data availability statement

The datasets presented in this study can be found in online repositories. The names of the repository/repositories and accession number(s) can be found in the article/**Supplementary Material**.

## Author contributions

CB, FC undertook the bioinformatic analyses. HN and AS supervised the research and obtained the funding. AS and CB wrote the manuscript with input from all the authors. All authors contributed to the article and approved the submitted version.

## Supplementary material

Supplementary Table 1 DEGs, orthoDEGs, and scoDEGs from mice and humans in response to CHIKV infection.

Supplementary Table 2 Reciprocal Gene Set Enrichment Analysis.

Supplementary Table 3 ImmuneSigDB Gene Set Enrichment Analysis.

Supplementary Table 4 Upstream Regulator annotations enriched in DEG lists.

Supplementary Table 5 Information relating to three non-CHIKV human inflammatory disease datasets
