# Supplementary material for: Chikungunya patient transcriptional signatures faithfully recapitulated in a C57BL/6J mouse model
Source: Front Immunol. 2022 Dec 12;13:1092370. doi: 10.3389/fimmu.2022.1092370 (PMC9791225; doi:10.3389/fimmu.2022.1092370)
Supplement: Supplementary file 6 [file Presentation_1.pdf]

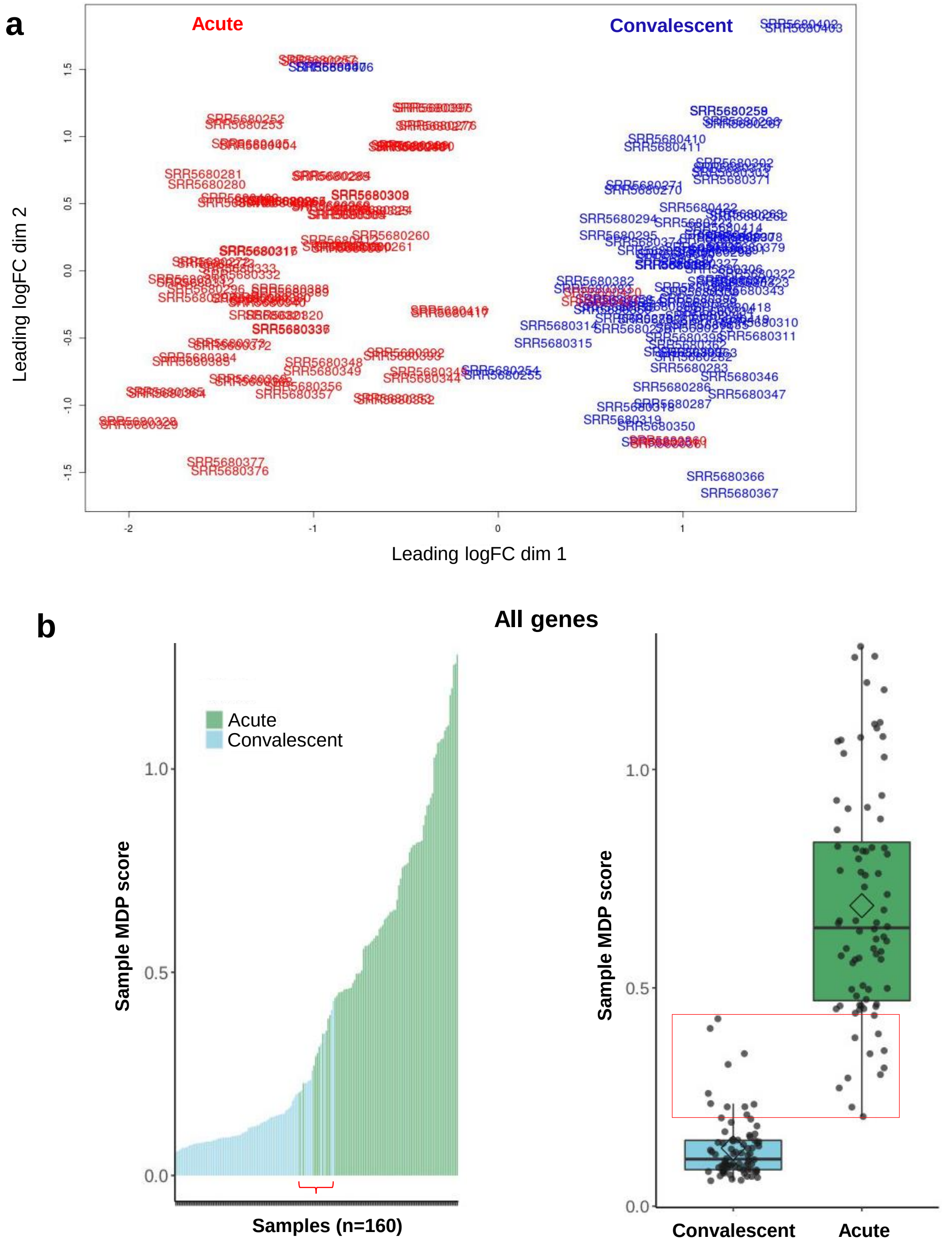

Fig. S1. Data set from PBMC of CHIKV infected Children. **a** MDS plot of paediatric samples from EdgeR analysis. Red: Samples from acute infection phase; Blue: Samples from convalescent phase. Samples SRR5680360, SRR5680361, SRR5680362, SRR5680363, SRR5680404, SRR5680405, SRR5680406, SRR5680407, SRR5680420, SRR5680421, SRR5680422, SRR5680423 belonging to patients 28, 39 and 43 clustered with samples of the opposite group (acute vs. convalescent), and were removed from further analyses. **b** From the Molecular Degree of Perturbation (MDP) (https://mdp.sysbio.tools/about) of 160 samples from paediatric dataset, it is possible to identify 10 samples from the acute phase and 10 samples from the convalescent phase with similar perturbation scores (red bracket and box). These samples were also removed from further analyses.

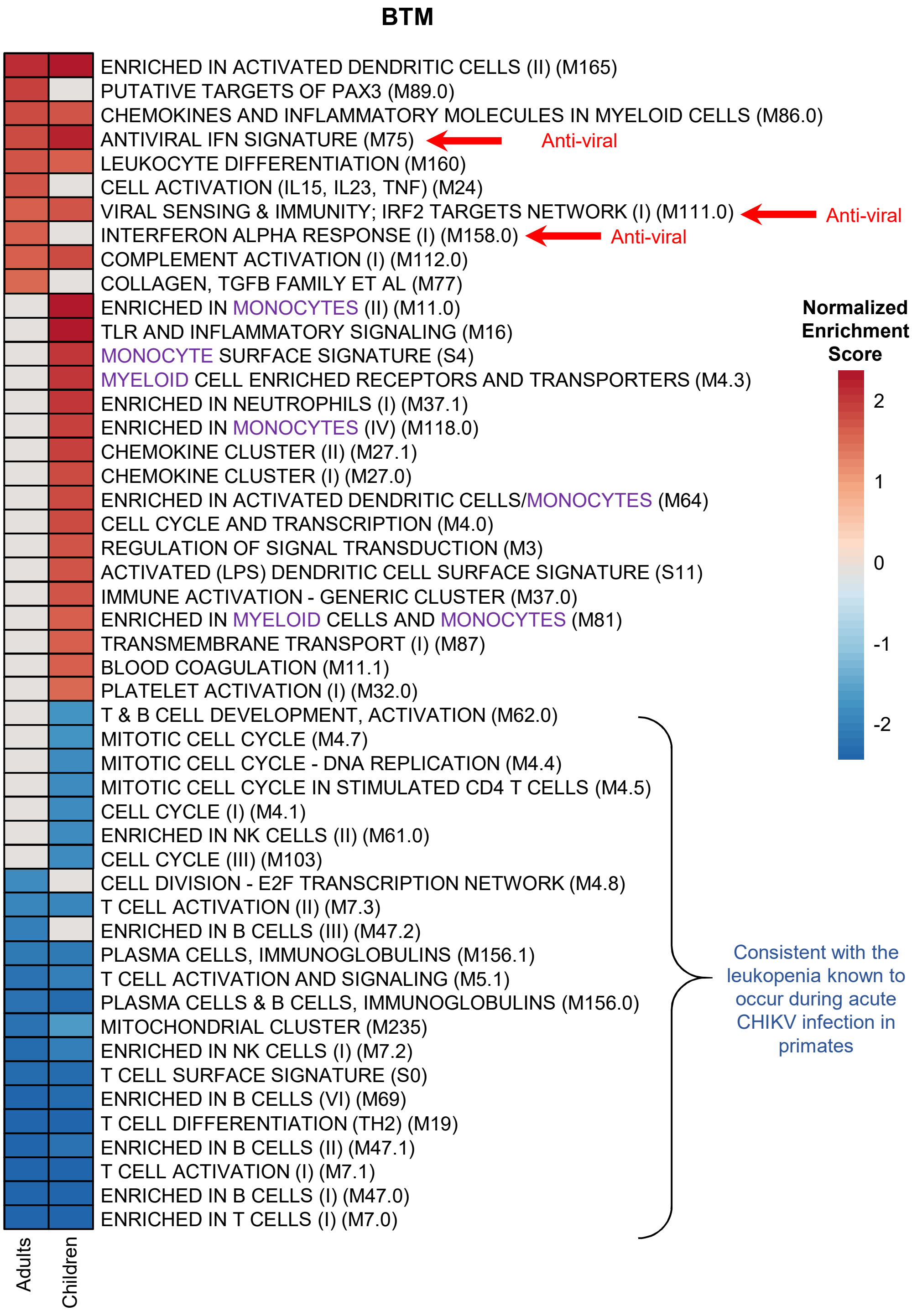

Fig. S2. Blood Transcription Modules (BTMs). Significant (q<0.05) GSEAs using BTMs and the gene lists from Adults and Children ranked by fold change. Negative NES (Blue) indicated the enrichment occurred in the down-regulated genes consistent with CHIKV-associated lymphopenia. Red arrows indicate shared anti-viral BTMs.

a

Groups	DEG type	With no HUGO ID (n)
Adults & Children	non-scoDEG	2171
	scoDEG	2
Mice	scoDEG	0
	non-scoDEG	3

Group	non-scoDEG (n)	With no HUGO ID (n)	Percent no HUGO ID
Adults	2662	1019	38%
Children	3071	1152	37%
		Sum = 2171	

b

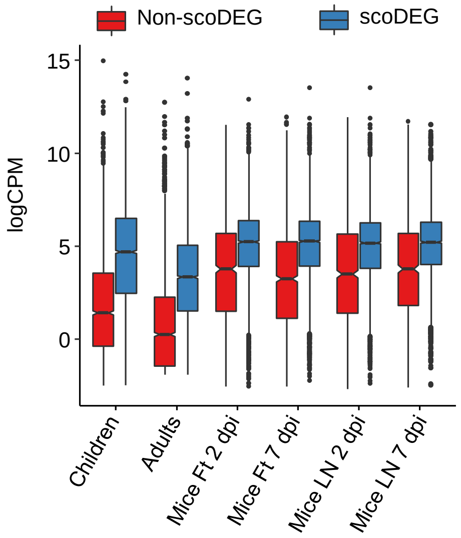

c

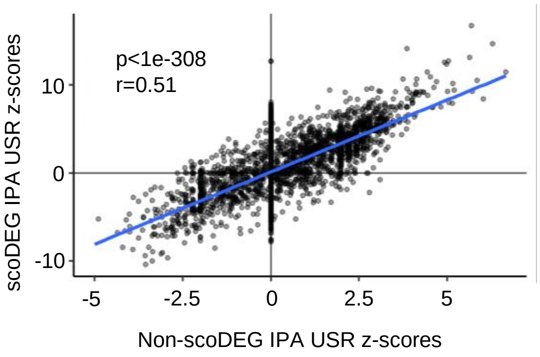

Fig. S3. Human DEGs that were not scoDEGs. From Fig. 1d about a third of human DEGs were not scoDEGs. **a** The human groups had a high number of DEGs with no HUGO ID, whereas this was rare for the Mice DEGs. Of these human non-scoDEGs, 38% and 37% lacked a HUGO ID (but have an Ensembl ID) for the Adults and Childrens groups, respectively (and primarily represent long non-coding RNA and pseudogenes). **b** The non-scoDEGs had much lower read counts than scoDEGs. **c** Pearson correlation between z-scores for IPA USRs obtained for human non-scoDEGs versus scoDEGs.

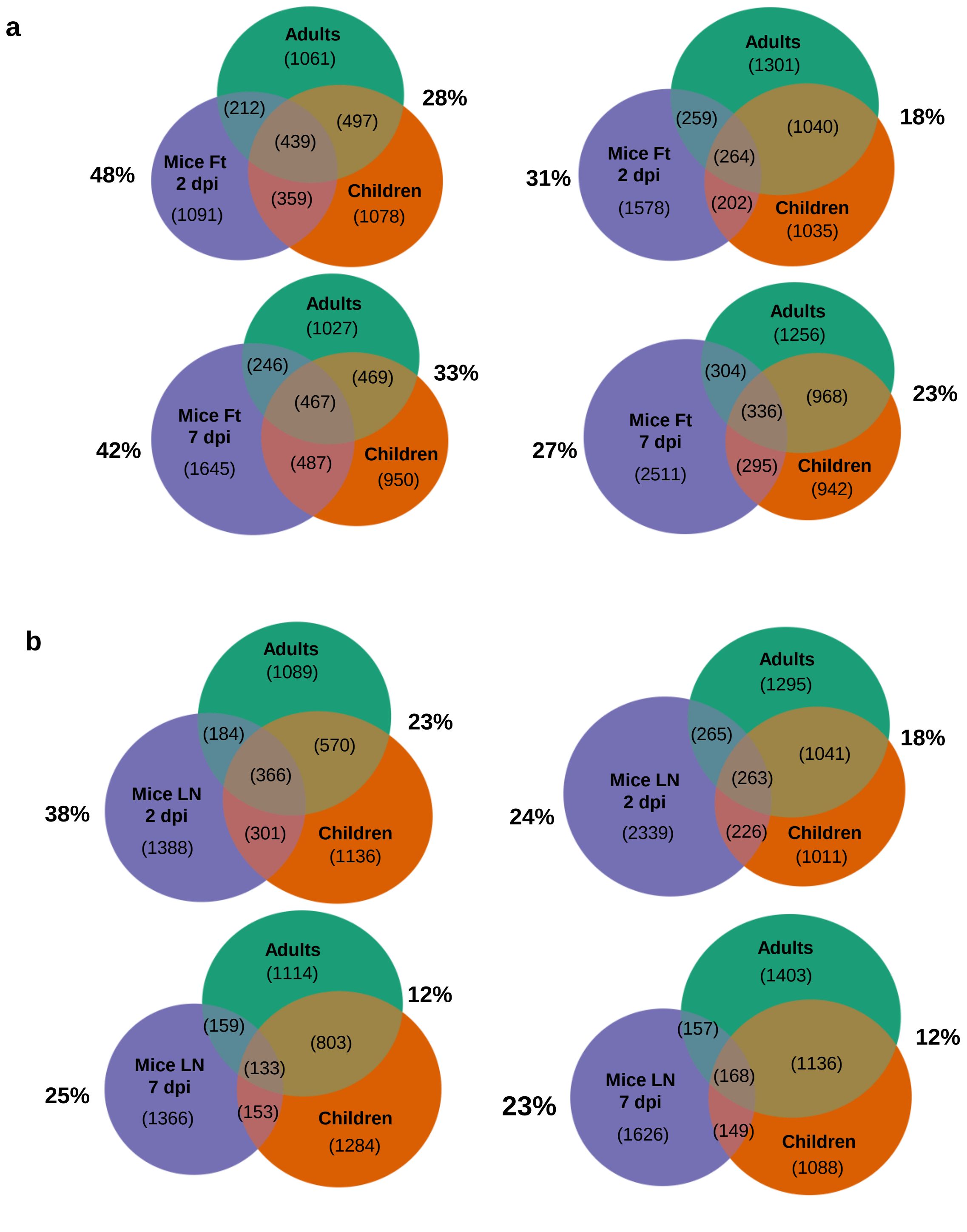

Fig S4. Euler diagrams showing scoDEG overlaps between mice and human groups. Using scoDEG lists from Fig. 1d. to provide the percentage overlaps shown in Fig. 2b. **a** Overlaps of scoDEGs for mice feet groups and Adults and Children.
b Overlaps of scoDEGs for mice LN groups and Adults and Children.

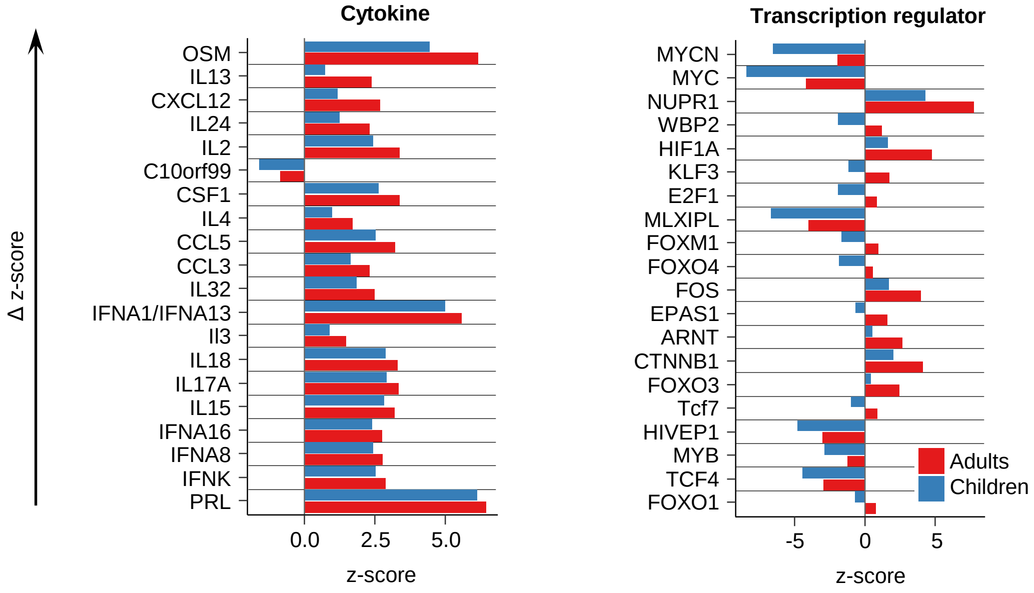

Fig. S5. Differences in IPA USR z-scores for Adults versus Children. DEGs obtained from peripheral blood of acutely CHIKV-infected Adults and Children were separately analysed by IPA and Cytokine and Transcription regulator USRs ranked by differences in z-scores. A high level of concordance between Adults and Children is apparent, with differences in z-scores often low, and where z-scores are positive for one and negative for another, the z-scores are low.

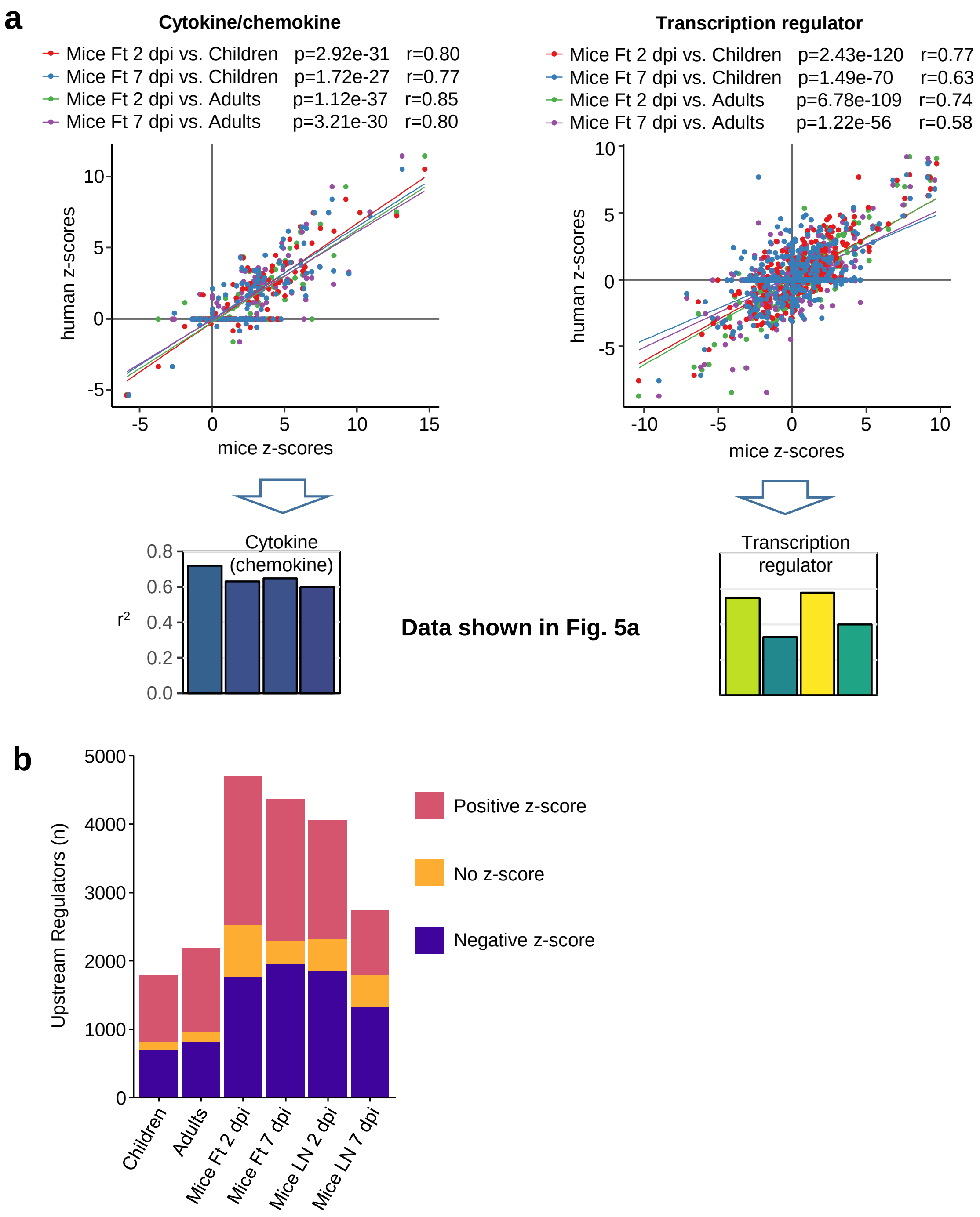

Fig. S6. a Examples of Pearson correlation plots for selected data from Fig. 5a. Graphical representation of how the data for the bar charts shown in Fig. 5a were obtained. **b Different number of USRs for mice and human.** DEG lists from each group (Fig. 1d) were analysed by IPA using the USR feature. IPA identified ≈2 fold more significant ($p<0.05$) USRs in the mice groups. This may reflect a murine bias in the IPA USR annotations. Where a USR is present in mice and absent in humans, a z-score value of 0 was given to the latter. The disparity in USR numbers, despite similar DEG numbers, would likely influence the correlations shown in a and Fig. 5, and arguably could result in an under-estimation of the level of congruence.

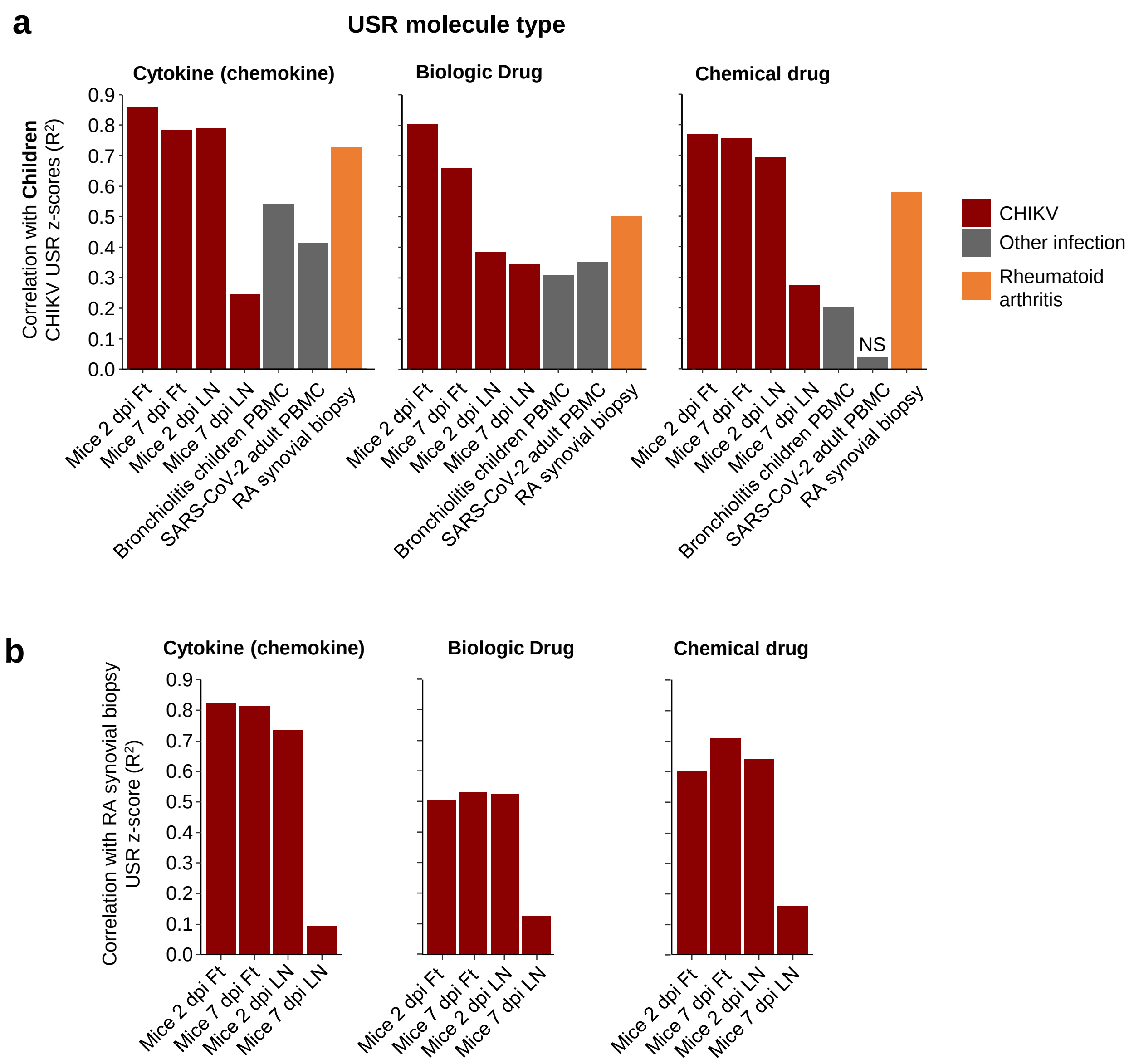

Fig. S7. IPA USR z-score correlations for CHIKV infected Children and for RA. a As for Figure 6a but using z-scores of USRs from peripheral blood of CHIKV-infected Children. **b** As above but comparing rheumatoid arthritis (RA) with mice groups.

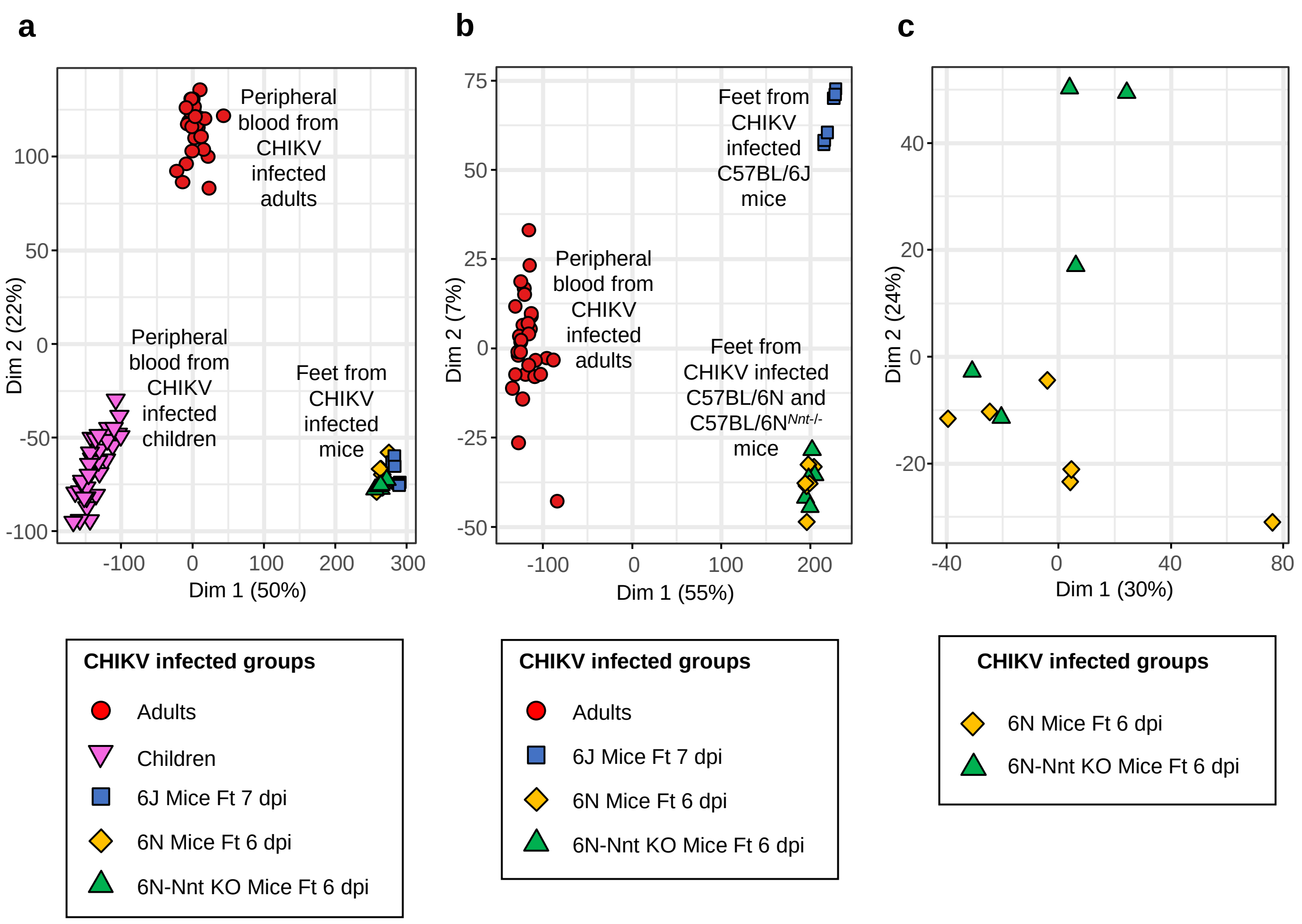

Fig. S8. MDS plots for CHIKV-infected samples for Adults, Children, C57BL/6J and C57BL/6N mice. a MDS using orthologous genes for infected feet (peak arthritis) for C57BL/6J and C57BL/6N mice, and peripheral blood from acutely infected adults and children. **b** MDS using orthologous genes for infected feet (peak arthritis) for C57BL/6J and C57BL/6N mice, and peripheral blood from acutely infected Adults. Removal of Children shows segregation of C57BL/6J and C57BL/6N mice. **c** Although no segregation is seen for C57BL/6N and C57BL/6N$^{Nnt-/-}$ mice in (a) and (b) where human samples are included, segregation is observed when they are analyzed by themselves. C57BL/6J and C57BL/6N mice have a number of genetic differences including *Nnt* (partially deleted and not functional in C57BL/6J mice). *Nnt* and other genes that differ between C57BL/6J and C57BL/6N result in ameliorated foot swelling in C57BL/6N mice (Rawle et al 2022).
